# A cluster randomized controlled cross-over bed net acceptability and preference trial in Solomon Islands: community participation in shaping policy for malaria elimination

**DOI:** 10.1186/1475-2875-8-298

**Published:** 2009-12-16

**Authors:** Jo-An Atkinson, Albino Bobogare, Andrew Vallely, Leonard Boaz, Gerard Kelly, William Basifiri, Simon Forsyth, Peter Baker, Bridget Appleyard, Hilson Toaliu, Gail Williams

**Affiliations:** 1 Pacific Malaria Initiative Support Centre, Australian Centre for International and Tropical Health, School of Population Health, University of Queensland, Brisbane, Australia; 2 National Vector Borne Disease Control Program, Ministry of Health, Honiara, Solomon Islands; 3 Save the Children Australia, Port Vila, Vanuatu

## Abstract

**Background:**

A key component of the malaria elimination strategy in Solomon Islands (SI) is widespread coverage of long-lasting insecticidal nets (LLINs). The success of this strategy is dependent on LLIN acceptability and compliance. There has been unresolved debate among policy makers and donors as to which type of LLIN would be most appropriate for large-scale distribution in SI, and anecdotal reports of a lack of acceptability of certain brands of LLINs. A cluster randomized controlled crossover bed net acceptability and preference trial was therefore carried out from July to September, 2008 to inform policy and to facilitate community engagement and participation in the selection of the most appropriate LLIN for use in SI.

**Method:**

A three-stage sampling method was used to randomly select the study population from Malaita Province, SI. Three brands of LLINs were assessed in this study: Olyset^®^, PermaNet^® ^and DuraNet^®^. Bed net acceptability and preference were evaluated through surveys at three defined time points after short and longer-term trial of each LLIN.

**Results:**

The acceptability of PermaNet^® ^after short-term use (96.5%) was significantly greater than Olyset^® ^(67.3%, *p < 0.001*) and DuraNet^® ^(69.8%, *p < 0.001*). The acceptability of DuraNet^® ^and Olyset^® ^after short-term use was not significantly different at the 5% level. LLINs that were perceived not to prevent mosquito bites were significantly less acceptable than LLINs that were perceived to prevent mosquito bites (OR 0.15; 95%CI 0.03 to 0.6). LLINs that allow a pleasant night's sleep (OR 6.3; 95%CI:3.3-12.3) and have a soft texture (OR 5.7; 95%CI:1.9-20.5) were considered more acceptable than those that did not. Olyset^®^'s acceptability decreased over time and this was due to net wrinkling/shrinkage after washing resulting in reduced efficiency in preventing mosquito bites. The increase in DuraNet^® ^acceptability was a result of a reduction in minor adverse events following longer-term use.

**Conclusion:**

This research was conducted to inform LLIN procurement as part of the national malaria control and elimination programme in SI. The success of malaria elimination in the Pacific and elsewhere relies on provision of acceptable interventions, consideration of local-level realities and engagement of communities in strategy development.

**Trial Registrations:**

Clinical trials ACTRN12608000322336

## Background

Community engagement and participation has played a critical role in successful disease control and elimination campaigns in many countries [1-5]. Participation can be difficult due to the complexity of cultural, social, and practical issues that affect a population's behaviour; their perceptions of health and disease priorities; the acceptability of particular interventions and the process by which communities are engaged in strategy development [6-12].

In March 2008, the Solomon Islands (SI) and Vanuatu governments elevated the goal of their National Malaria Programmes (NMPs) to elimination, with one province in each country targeted for early success by 2014. In Solomon Islands, this province is Temotu. Key vector control components of the elimination strategy in Temotu are widespread coverage and use of long-lasting insecticidal nets (LLINs) and targeted indoor residual spraying (IRS). Although the technologies of these interventions have improved, their success is still contingent on their acceptability and compliance at the household and community level. A previous study in SI reported that only 52% of the sampled households comply with year round use of bed nets, indicating that considerable work remains to be done to achieve meaningful community engagement and participation in the widespread uptake of this intervention if elimination of malaria is to be feasible [13].

Different brands of LLINs can have dissimilar levels of acceptability based on their physical characteristics and perceived effectiveness in preventing mosquito bites and malaria [14]. Providing populations with LLINs they find most acceptable, and accompanying LLIN distribution with behaviour change communication that addresses the misconceptions and behavioural factors that impact on their use, will be vital to achieving high coverage and sustained use of LLINs.

LLINs, specifically Olyset^® ^(polyethylene) nets, were introduced in SI in 2003. However, over the following years anecdotal reports indicated that these nets were not popular due to their stiff texture and tendency to wrinkle and shorten after washing (making them difficult to fold under woven palm sleeping mats). They were also reportedly disliked because of their large mesh size that was perceived to allow mosquitoes to penetrate the net. A study in Tanzania similarly reported that despite Olyset^® ^nets being appreciated for their durability, they were reported to be too small in size compared to the ordinary polyester nets being used by the population since the late 1990's, and were perceived as having a mesh size that could allow entry of mosquitoes. In addition, participants estimated that the effectiveness of new Olyset^® ^LLINs in repelling mosquitoes was lost within 6-24 months [15].

PermaNet^® ^(polyester) bed nets were introduced in SI in 2007. However, anecdotal reports soon emerged that although these nets were popular, they had reduced ventilation compared to Olyset^® ^LLINs, their longevity in field conditions may be less than desirable and, consequently, they would require more frequent replacement. The use of two brands of LLINs in SI with very different physical qualities and washing instructions created challenges for distribution and education.

The malaria elimination strategy in SI calls for a significant scaling up of coverage of LLINs. Although there is agreement that a single brand of LLIN should be distributed, there has been much debate as to which type of LLIN would be most appropriate. Providing a net that is more durable would be most cost-effective in that it requires a less frequent replacement cycle. This would place fewer logistical demands on an already overstretched health system. However, distributing an LLIN that lacks acceptability in the target population may jeopardize the overall objectives of the programme. Suggestions that it would be adequate to impose an intervention on a population and accompany it with intensive education and social marketing without due regard for the issues surrounding its lack of acceptability, pay no heed to lessons learned from the malaria eradication era of the 1950's and '60's [4,16,17]

Results of a recent qualitative inquiry in Temotu and Malaita Provinces, Sl, were in agreement with anecdotal reports of Olyset^®^'s limited acceptability in Solomon Islands, primarily due to a perceived failure to adequately prevent mosquito bites [14]. Protection from mosquito bites has been reported in a number of studies as a key determinant of bed net use [13,18,19]. Olyset^®^'s lack of acceptability in both central Malaita Province and the more remote Temotu Province warranted further investigation prior to large-scale procurement and distribution of LLINs in SI. A cluster randomized bed net acceptability and preference trial was, therefore, undertaken by the Vector Borne Disease Control Programme (VBDCP), SI and the Pacific Malaria Initiative Support Centre (PacMISC) to inform policy and to facilitate community participation in the selection of the most appropriate brand of LLIN for distribution in SI. The trial included the assessment of a third type of LLIN (DuraNet^®^), which has interim approval by the WHO Pesticide Evaluation Scheme (WHOPES), and which shares some of the characteristics of both PermaNet^® ^and Olyset^® ^(Table 1).

**Table 1:** LLIN characteristics

BRAND	MATERIAL	INSECTICIDE	MESH SIZE	FIBER THICKNESS
Olyset (Sumitomo Chemical Company, Japan)	Polyethylene	1,000 mg/m^2^ permethrin	4 × 4 mm	150 denier

PermaNet 2.0 (Vestergaard-Frandsen, Denmark)	Polyester	55 mg/m^2^ deltamethrin	1.5 × 1.5 mm	100 denier

DuraNet (Clarke Mosquito Control, USA)	Polyethylene	261 mg/m^2^ alphacypermethrin	2 × 2.5 mm	145 denier

## Methods

### Study area and target population

**Figure 1:**
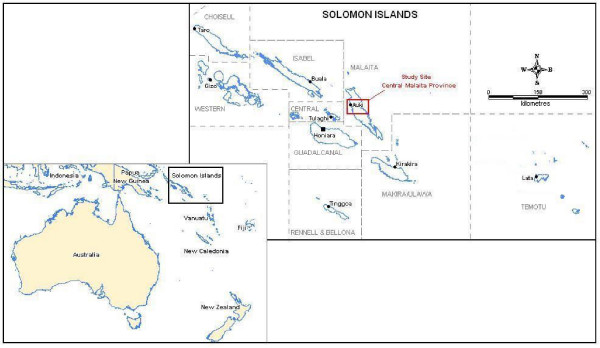
**Map of region showing study area**.

The trial was conducted from July to September 2008 in Malaita Province, Solomon Islands (Figure 1). The remoteness of Temotu, and an earlier qualitative study that showed agreement on the main issues of LLIN acceptability, preference and compliance between communities in Malaita and Temotu Provinces, suggested it would be acceptable to carry out this study in the more accessible Province of Malaita [14]. The study population consisted of heads of households and primary caregivers.

### Sample size

Sample size calculations were based on having 80% power to detect a 20% group difference in preference and/or acceptability (e.g. between 75-95%) at the 5% statistical level and suggested that a minimum of 90 households be recruited to this study. In order to take account of possible losses to follow-up, a 10-15% increase in sample size was recommended i.e. 102 households in total; 17 per trial group. This study recruited 105 households with 208 participants. The sample size was deemed to be adequate to measure bed net acceptability and preference in the study population with sufficient confidence.

### Study population

**Figure 2:**
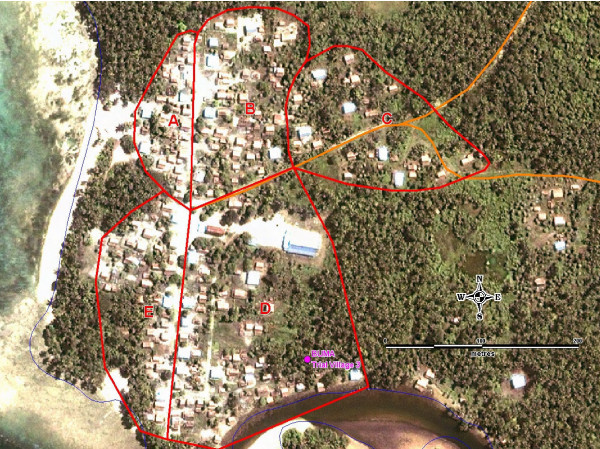
**Satellite imagery used for cluster randomisation in Buma village, Malaita Province**.

Three-stage sampling was used to select the study population. First, six villages in central Malaita Province, SI, were randomly selected from a list of villages with a population > 200 people. Second, each of the selected villages was randomly allocated to one of the 6 bed net trial groups. Finally, satellite imagery was used to identify natural clusters of households within each of the selected villages that had greater than 17-20 households. These clusters were verified in the field using a Global Positioning System (GPS) device (Figure 2). One cluster was then randomly selected within each village to be invited to participate in the study. This method was employed to increase chances of representativeness and to prevent uncomfortable exclusion of the immediate neighbours of households selected to participate in the study and receive free bed nets. All randomization was carried out using Excel generated random number tables.

**Figure 3:**
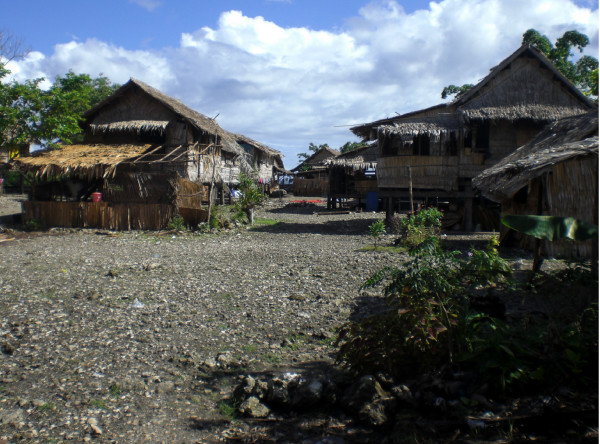
**Coastal village in Malaita Province showing typical housing composition**.

Housing in the study villages is predominately traditional with thatched rooves (Figure 3). Woven palm sleeping mats are used by most of the rural population. Melanesian languages are spoken throughout the Province and while English is the official language, the lingua franca is Solomon Islands Pidgin.

### Procedure

Baseline information was collected at study entry from household heads and primary caregivers which captured information on participant demographics, factors affecting human-vector contact including bed net use, and pre-existing bed net preferences. Data was entered directly into handheld PDA/GPS devices at the time of participant interview.

Each household initially received one of three LLIN types: PermaNet^®^, DuraNet^® ^or Olyset^®^, which was determined by random assignment. Each household was given sufficient LLINs to ensure coverage for all household members (including children). Participants were asked to air their new bed nets prior to use according to manufacturer's instructions.

Following 10 days of LLIN use, field staff re-visited each household, administered a short acceptability questionnaire to the household head and primary caregiver, gathered data on adverse events and distributed a second LLIN type as specified in the study allocation list (see Additional file 1: overview of cross-over study design).

After a further 10-day period this process was repeated and in addition, the head of household and primary caregiver were asked to state which of the two LLINs used they preferred. Households were then asked to continue using the LLINs given at second allocation for a further three weeks and to wash their nets at least once during this period. This was to ensure that LLIN acceptability and preference were not based on the characteristics of a new net but reflected field conditions/actual conditions of use. A final visit was carried out after this additional three-week period to re-assess acceptability, preference and the impact of washing and prolonged usage on these variables.

### Ethical aspects

This research was approved by the National Health Research Ethics Committee, Solomon Islands and the Medical Research Ethics Committee, University of Queensland, Australia. The trial was registered with the Australian and New Zealand Clinical Trial Register (ACTRN12608000322336). Individual informed consent (written or witnessed thumb print) was obtained from all participants prior to the trial following a verbal and written explanation of study aims and procedures.

### Statistical analysis

Questionnaire data were downloaded from PDAs, converted to Microsoft Access and checked for consistency with FLG validation scripts [20]. Computation and data handling were carried out using SAS 9.2 (SAS Institute Inc., NC, USA) and R 2.9.1 (Development Core Team R, Vienna, Austria) [21,22]. Simple proportions were employed to describe study parameters and multivariate analysis carried out to explore determinants of LLIN acceptability and preference with appropriate adjustment for a cross-over design.

Preliminary data analysis consisted of χ^2 ^(chi squared) tests and logistic regressions in order to examine relationships between acceptability, preference and other questionnaire data. Separate standard crossover analyses for binary data were performed on head of households and primary caregivers over both short- and longer-term bed net trial periods. McNemar's tests for differences in outcomes between household heads and primary caregivers were not significant at the 5% level. Differences in acceptability between the three types of bed nets were assessed by analysis of binary cross-over data as outlined in Jones and Kenward (2003) [23]. Carryover effects, village and household effects were included in the models. Since carryover effects were not estimable for preference data, preferences were analysed by χ^2 ^(chi squared) tests.

Finally, two techniques were employed to assess the effects of other factors such as perceptions about the nets and adverse reactions on bed net acceptability. Firstly, logistic regression was used to choose a final model by stepwise backwards (and forwards) selection based on the Akaike Information Criterion (AIC) for inclusion of factors [24]. Secondly, as a non parametric alternative, recursive partitioning via classification trees were employed [25]. The commonly used *Gini *index was employed to obtain the final pruned tree [24,25].

## Results

### Baseline demographics

**Figure 4:**
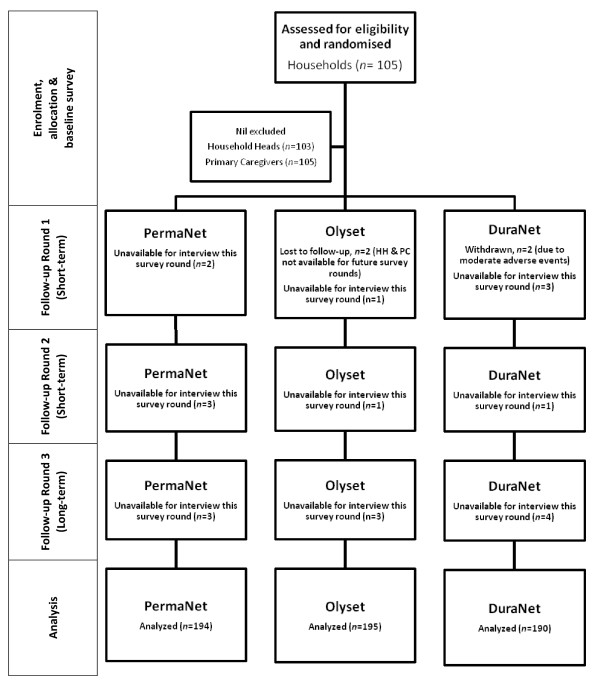
**Study profile (participant flow)**.

In all, 105 households were recruited to the study [household heads (n = 103); primary caregivers (n = 105)]. One household was lost to follow-up and another withdrawn due to moderate adverse reactions to DuraNet^® ^bed nets in Round 1 of the trial. Therefore, 103 households completed the study [household heads (n = 101); primary caregivers (n = 103)] (Figure 4).

There were no significant differences between the six trial villages in age, gender, level of education and household size (Table 2). Employment status and occupation varied between villages, but were controlled for in the carry-over analyses. Household heads (HH) were universally male and primary caregivers (PC) were female. The mean age of HH's was 42 years and PC's 38 years. 29.1% of primary caregivers compared to 14.6% of household heads had received no formal education; 57.4% of HHs compared to 33.9% of PCs had completed primary education or above. 60% of HHs and 68% of PCs had no formal employment hence many relied on subsistence farming or fishing. Household heads and primary caregivers differed significantly in age (*p *= 0.0298), level of education (*p *= 0.0198) and type of occupation (*p *< 0.001).

**Table 2:** Baseline demographics of participants by trial village.

Characteristic	Buma (*n *= 34)	Kwaisuliniu (*n *= 34)	Ngadaifiu (*n *= 34)	Ngalisagore (*n *= 33)	Oibola (*n *= 36)	Radefasu (*n *= 35)	*p*-value
**Mean age**: *n *± SD	36.5 ± 11.0	42.7 ± 12.2	40.4 ± 13.6	40.6 ± 14.3	40.9 ± 14.0	40.1 ± 14.0	0.550

**Gender**: *n *(%)							
Males	17 (50)	17 (50)	17 (50)	15 (45.5)	18 (50)	18 (51.4)	0.998
Females	17 (50)	17 (50)	17 (50)	18 (54.5)	18 (50)	17 (48.6)	

**Education**: *n *(%)							
No education	13 (38.2)	9 (26.5)	8 (23.6)	6 (18.2)	3 (8.3)	6 (17.2)	0.397
Primary only	14 (41.2)	20 (58.8)	18 (52.9)	20 (60.6)	26 (72.2)	23 (65.7)	
Secondary	7 (20.6)	4 (11.8)	7 (20.6)	6 (18.2)	5 (13.9)	4 (11.4)	
Higher education	0	1 (2.9)	1 (2.9)	1 (3.0)	2 (5.6)	2 (5.7)	

**Mean household size**: *n *± SD	6.8 ± 2.3	7.5 ± 3.1	5.2 ± 2.0	5.8 ± 2.9	5.5 ± 1.8	6.0 ± 2.6	0.07

**Employment**: *n *(%)							
Full-time	1 (2.9)	0	1 (2.9)	5 (15.2)	15 (41.7)	27 (77.1)	< 0.001
Part-time/casual	1 (2.9)	3 (8.8)	2 (5.9)	8 (24.2)	8 (22.2)	1 (2.9)	
Not employed	32 (94.2)	31 (91.2)	31 (91.2)	20 (60.6)	13 (36.1)	7 (20.0)	

**Occupation**: *n *(%)							
Farming/agriculture	25 (78.1)	14 (53.9)	3 (12.5)	9 (28.1)	21 (58.2)	16 (51.6)	< 0.001
Domestic duties	4 (12.5)	5 (19.1)	5 (20.8)	13 (40.6)	1 (2.8)	3 (9.7)	
Trade (carpenter)	1 (3.1)	1 (3.9)	4 (16.7)	3 (9.4)	1 (2.8)	3 (9.7)	
Professional/Church elder	2 (6.3)	6 (23.1)	11 (45.8)	7 (21.9)	11 (30.6)	9 (29.0)	
Student	0	0	1(4.2)	0	2 (5.6)	0	

Despite the demographic differences between household heads and primary caregivers there were no significant differences in their attitudes towards the acceptability of the different brands of LLINs trialled, with the exception of Olyset^® ^in the first survey round. In this first round 93% of household heads found Olyset to be acceptable compared to 59% of primary caregivers. There were no significant differences between household heads and primary caregivers with regards to their preferences for LLIN type, size, shape or colour, nor with a number of factors affecting human vector contact. Therefore, to avoid repetition, where the responses of household heads and primary caregivers do not differ significantly they have been combined in the results presented.

### Bed net acceptability

#### Short-term trial period (10 days)

After short-term trial of the nets, the acceptability of PermaNet^® ^(96.5%) was significantly greater than Olyset^® ^(67.3%, *p < 0.001) *and DuraNet^® ^(69.8%, *p < 0.001) *bed nets. The acceptability of DuraNet and Olyset was not significantly different at the 5% level.

#### Longer-term trial period (5 weeks)

**Figure 5:**
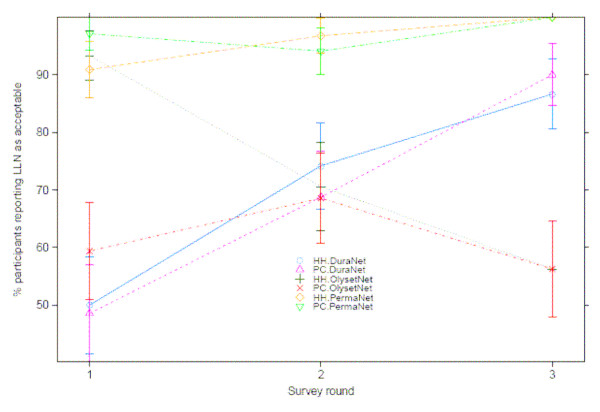
**Acceptability of Duranet^®^, Olyset^® ^and Permanet^® ^LLINs by survey round and gender**.

The acceptability of PermaNet^® ^(100%) remained significantly greater than Olyset^® ^(56.0%, *p < 0.001*) and DuraNet^® ^(88.5%, *p < 0.001*). In addition, the acceptability of Olyset^® ^was significantly lower than DuraNet^® ^(*p *< 0.001) after longer-term trial of the nets (Figure 5).

#### Changing acceptability between short- and longer-term trial periods

PermaNet^® ^acceptability remained high throughout the trial. Olyset^®^'s acceptability decreased by 17% after longer-term use of the net. Analysis using logistic regression and recursive partitioning (using classification trees) suggests that this decreased acceptability was due to wrinkling of the net over time and its reduced efficiency in preventing mosquito bites. DuraNet^® ^acceptability increased by over 40% between short- and longer-term trial periods as a result of the decreased incidence of side effects of a new net over time. There were no significant differences in frequency of washing of the trial nets between the three brands of LLINs.

#### Determinants of acceptability

LLINs that were perceived not to prevent mosquito bites were significantly less acceptable than LLINs that were perceived to prevent mosquito bites (OR 0.15; 95%CI 0.034 to 0.6). The LLINs that were reported to allow a pleasant night's sleep (OR 6.3; 95%CI 3.3 to 12.3) and have a soft texture (OR 5.7; 95%CI 1.9 to 20.5) were considered more acceptable for use than those that didn't. In addition, acceptability of LLINs was found to be negatively influenced by net wrinkling (p = 0.0003); the perception that the LLIN did not reduce insects inside the house (p = 0.047); or if the net was considered too hot to sleep under by children (p = 0.023). Acceptability of LLINs was positively influenced by the absence of an adverse reaction to the net by household members (as reported by primary caregivers) (p = 0.0002) or if the LLIN was perceived as durable (p = 0.0004).

**Figure 6:**
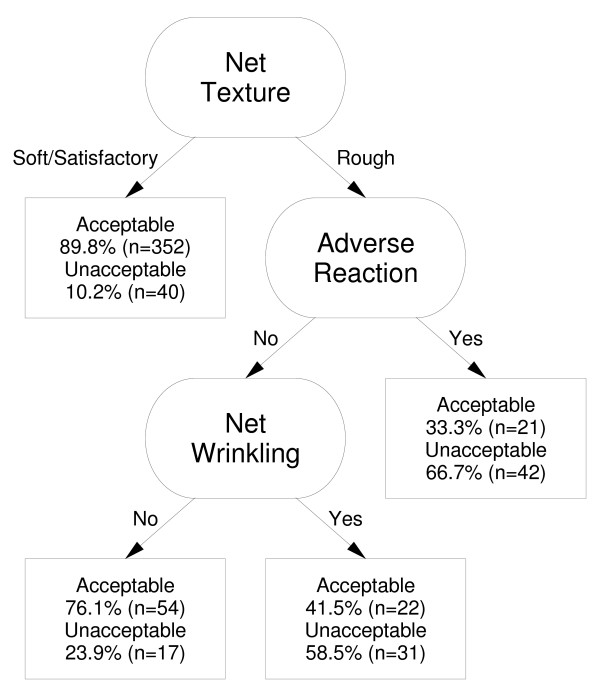
Pruned classification tree of variables associated with acceptability of LLINs over all three survey rounds*. * The ability of LLINs to provide a pleasant night's sleep was the primary determinant of acceptability. This variable was removed from the classification tree to avoid over fitting and reduce complexity of the figure. In addition, ability of the LLIN to provide a pleasant night's sleep is considered to be a product of the other remaining variables in the classification tree. † n = 579.

The hierarchy of variables associated with acceptability of LLINs over all three survey rounds is presented in Figure 6. This classification tree represents a hierarchy of the statistical strength of association between bed net characteristics and LLIN acceptability, with the most related factor for acceptability being at the top of the figure, e.g. if the LLIN was perceived to be soft or satisfactory, 89.8% of participants found it to be acceptable. If the LLIN was perceived to be rough and had an adverse reaction, only one-third of participants found the net to be acceptable.

#### Bed net preference

Overall, PermaNet^® ^was by far the preferred net with 75.4% of those who trialled it reporting it their net of choice for future use. Of the participants who trialled DuraNet^®^, 45% preferred it. However, only 23.9% of the participants who trialled Olyset^® ^identified it as their preferred net.

Perceived LLIN durability was not significantly correlated with bed net preference. Determining factors for LLIN preference over both short- and longer-term trial periods were if a particular net was considered better at preventing mosquito bites while sleeping (p < 0.0001); better at reducing insects in the house (p < 0.0001); allowed the most pleasant sleep (p = 0.0001); was available in appropriate sizes for the family (p = 0.0009); and was more attractive in appearance (p = 0.0005). With regards to size, shape and colour preferences (measured at baseline survey), there was a greater preference for larger bed nets (family size & extra large family size); darker colours (blue & green); and an almost equal preference for rectangle and cone shaped bed nets.

#### Factors influencing human vector contact

Compliance with personal protection and lifestyle factors play a significant role in human exposure to mosquitoes and hence vulnerability to malaria. The proportion of households with an outdoor kitchen was 93.3%. Over 44% of household heads and 37% of primary caregivers in this study reported that they are usually outside during or after sunset, which is a peak biting time for *Anopheles farauti*, the primary vector of malaria in Solomon Islands [26]. Although 92% of nets owned by participants were observed hanging within households, only 42% of household heads and 51% of primary caregivers reported that they slept under a bed net the previous night. The primary reasons given for bed net non-use by participants the previous night were; insufficient nets available in the household (46.4%); the nets are too hot to sleep under (14.3%); and that there were not many mosquitoes around (12.5%).

Bed nets were the most commonly reported method of mosquito protection used by participants (56%), however 27.6% of participants reported they did not use any protection methods and a further 32% use ineffective methods (i.e. fire/smoke, blankets, clothing, cleaning around house, coconut oil, mosquito swats). No participants reported using topical insect repellents. Of those participants that reported using bed nets, 32% indicated that they were used seasonally or intermittently and 68% reported year round use. There were no significant differences between household heads and primary caregivers in their reports of methods of mosquito protection used, and patterns of bed net use.

#### Adverse events

No severe adverse events were observed in this study. Two members of one household (a mother and her son) reported moderate adverse events (burning sensation of the eyes, itching and skin rashes and sores over their bodies) felt by investigators to be possibly related to DuraNet^® ^use and which required treatment. This household was subsequently withdrawn from the study.

Overall, there was a significant difference in adverse reactions between bed nets (p < 0.001), and an unexpectedly high proportion of minor adverse events reported by participants who trialled DuraNet^®^. Adverse reactions decreased over time (roughly linearly) for DuraNet^® ^and Olyset^®^. Adverse reactions to PermaNet^® ^were lower overall and did not differ significantly between survey rounds (Table 3).

**Table 3:** Proportion of households that had at least one minor adverse event.

Survey Round	DuraNet (%)	Olyset (%)	PermaNet (%)
**Round 1**: *(Short-term trial of LLINs)*	88.6	24.2	5.7

**Round 2**: *(Short-term trial of LLINs)*	56.3	5.7	3.0

**Round 3**: *(Longer-term trial of LLINs)*	16.7	0	0

The nature, severity and scope of adverse events reported during the trial were consistent with earlier studies and were anticipated by the investigators. The types of minor adverse reactions reported were headache, skin, eye and nose irritation and dizziness. The duration of symptoms in all cases was 1-3 days.

## Discussion

### LLIN acceptability and preference

This cluster randomized controlled cross-over trial confirms anecdotal reports and the findings of an earlier qualitative study which found that Olyset^® ^nets are less acceptable than PermaNet^® ^LLINs in Solomon Islands [14]. The main determinants of LLIN acceptability in this trial were found to be reduced protection from mosquito bites and ability to allow a pleasant night's sleep. Olyset^®^'s limited acceptability was due to wrinkling which causes the net to become untucked from the sleeping mat, allowing mosquitoes to enter and bite. PermaNet^® ^hangs straight and remains tucked beneath bedding throughout the night affording maximum protection from bites. Unlike Olyset^®^, this characteristic is unaffected by washing [14]. This may explain PermaNet^®^'s high acceptability and preference over Olyset^® ^LLINs.

A previous qualitative study in SI by our group found a notional preference for DuraNet^® ^as it appeared to represent the middle ground between the characteristics of Olyset^® ^and PermaNet^® ^LLINs [14]. This preliminary preference was not sustained in subsequent field trials of the net. The discomfort created by the high initial minor adverse event rate experienced by participants using DuraNet^® ^LLINs most likely contributed to its early lack of acceptability. There is no obvious explanation for the high incidence of minor adverse events in those trialling DuraNet^® ^bed nets. The side effects experienced are reported to be typical for pyrethroids [27,28], however, it is unusual that they were experienced by such a large proportion of participants trialling DuraNet compared to those trialling PermaNet and Olyset. Although these symptoms were minor and transient, there is an obvious risk that community support for this intervention will be jeopardized if it creates adverse events in a significant proportion of the population. It is unclear if DuraNet^®^'s acceptability and preference would approach that of PermaNet^® ^over a longer period of use or whether differences in bed net durability may influence relative acceptability in the medium to long term. These issues warrant further investigation.

### Implications for malaria elimination in Solomon Islands

The most commonly reported reason for non-use of bed nets by participants in the pre-trial survey was a lack of ownership of a net. This will be addressed during a national bed net distribution campaign in 2009-10. However, behaviour change communication will need to accompany this distribution to address vulnerability issues relating to seasonal or intermittent bed net use, ineffective mosquito prevention methods and lifestyle factors that increase human-vector contact. With high coverage and consistency of LLIN use such an important contributor to the success of malaria elimination, it is vital that acceptability issues be taken into account during program design implementation and monitoring and evaluation.

Although PermaNet^® ^was identified by this study as the most acceptable and preferred LLIN, community perceptions and opinion are not the only issues policy makers need to consider. LLIN longevity and effectiveness under field conditions, distribution costs and net replacement cycles vary so that policy makers may need to critically balance these against acceptability concerns.

### Limitations of the study

This study was carried out between July and September, which are the cooler months of the year. It is unclear what effect seasonal variations in evening temperature or humidity would have on the acceptability of the three brands of LLINs as a result of their varying mesh size and hence ability to provide ventilation. As discussed previously, there was a high proportion of minor adverse events reported by participants who trialled DuraNet^® ^in the first round. Therefore, at the direction of Medical Research Ethics Committee, University of Queensland, field researchers recommended that participants air new DuraNet^® ^bed nets out for 1-2 days beyond manufacturer's instructions in the second round (in an attempt to reduce the incidence of minor adverse events). It is unclear what impact (if any) this may have had on trial outcomes. It should also be noted that conclusions based on the outcome of this study cannot be generalized to elsewhere.

## Conclusions

Providing populations with LLINs they find most acceptable, and accompanying their distribution with behaviour change communication initiatives that address behavioural factors that impact on their use, is essential for achieving the high coverage of LLINs required for malaria elimination. In undertaking operational research on the acceptability of this key intervention, Solomon Islands have acknowledged the importance of community consultation in informing its strategy for malaria elimination. Further evidence of the Solomon Islands commitment to meaningful community engagement and participation are their plans for further action-orientated research and participatory methods to engage communities in the development of behaviour change communication strategies and to explore perceptions and acceptability of other key interventions such as indoor residual spraying (IRS), rapid diagnostic tests (RDTs) and artemisinin-based combination therapy (ACT). It is vital that such operational research be fully integrated within programme design, implementation and monitoring and evaluation if malaria elimination is to be achieved in Solomon Islands and elsewhere.

## Competing interests

Olyset^® ^and DuraNet^® ^LLINs were supplied by their respective distributors free-of-charge for use in this trial. PermaNet^® ^bed nets were already available in-country. However, the authors declare that they have no competing interests.

## Authors' contributions

Authors that participated in the conception of study design were AB, AV, LB, GW, JA, HT & BA. Training of field researchers in quantitative methods and logistics was carried out by JA, GK & LB. The field research activities were supported by GK, AB, LB, WB, JA & AV. Data conversion, cleaning and logical checks was carried out by SF & JA. Data analysis was carried out by PB and GW. Manuscript drafting was carried out by JA with support and contributions from all authors.

## Supplementary Material

Additional file 1 Overview of crossover study design. Click here for file
