# Supplementary figures and images for: A cluster randomized controlled cross-over bed net acceptability and preference trial in Solomon Islands: community participation in shaping policy for malaria elimination
Source: Malar J. 2009 Dec 16;8:298. doi: 10.1186/1475-2875-8-298 (PMC2803192; doi:10.1186/1475-2875-8-298)

**Additional file 1: Overview of crossover study design**

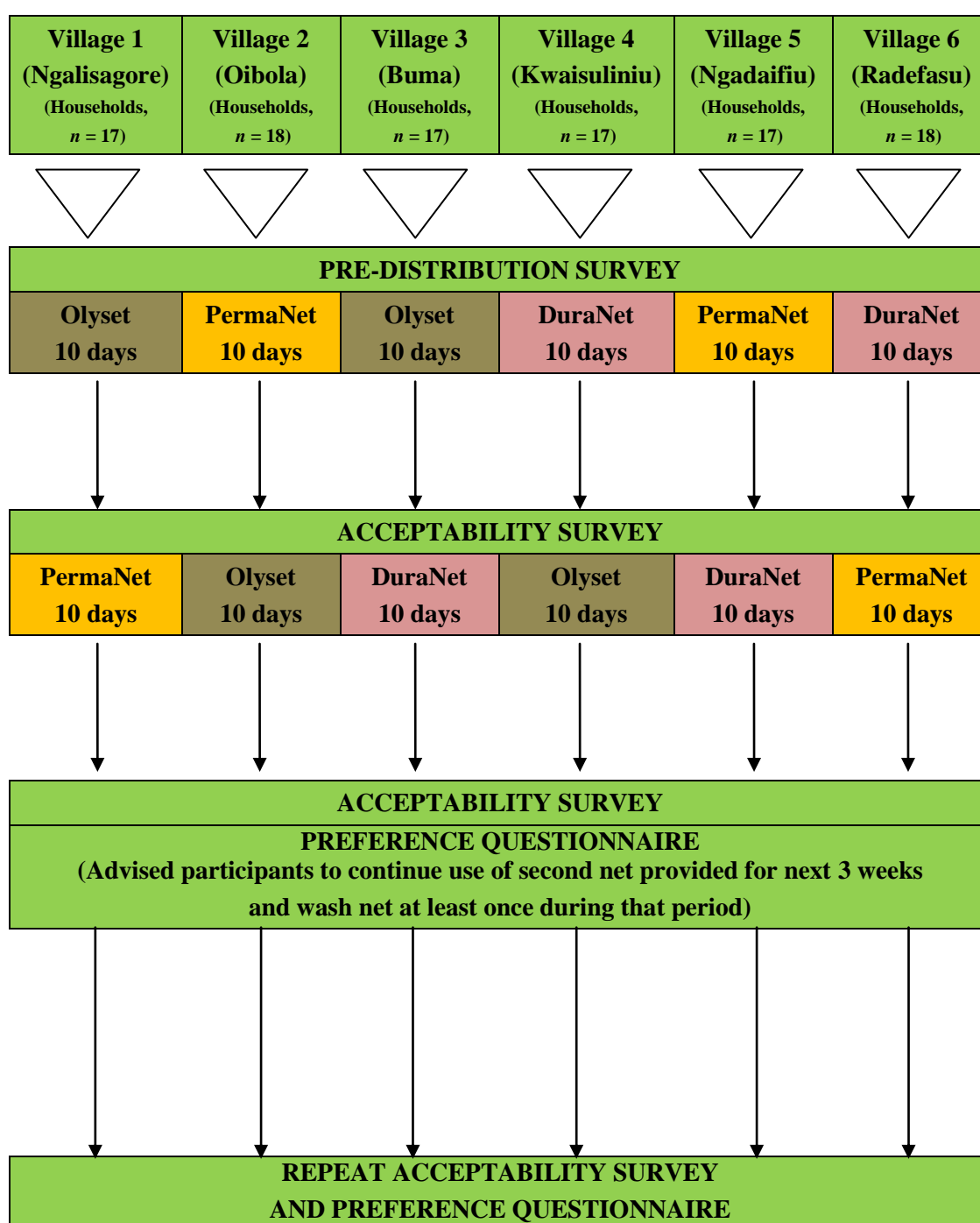

Supplement: Additional file 1 — Overview of crossover study design. [file 1475-2875-8-298-S1.PDF]
